# Corynebacterium striatum-Induced Native Valve Infective Endocarditis in an Immunocompetent Patient

**DOI:** 10.7759/cureus.67951

**Published:** 2024-08-27

**Authors:** Bernard P Parrish, Joseph Young, Mina M Benjamin, Nongnooch Poowanawittayakom

**Affiliations:** 1 Internal Medicine, Saint Louis University School of Medicine, St. Louis, USA; 2 Internal Medicine, SSM Health Saint Louis University Hospital, St. Louis, USA; 3 Cardiology, SSM Health Saint Louis University Hospital, St. Louis, USA; 4 Infectious Disease, SSM Health Saint Louis University Hospital, St. Louis, USA

**Keywords:** corynebacterium striatum, corynebacterium, infectious disease, central line-associated infections (clabsi), infective endocarditis

## Abstract

*Corynebacterium striatum* is often considered a contaminant in blood cultures due to often being found colonizing skin and mucous membranes. This case displays *C. striatum* infective endocarditis (IE) identified in an immunocompetent patient on a native valve. Despite treatment with vancomycin, the case was complicated by embolic infarcts to the spleen and left cerebellum along with the development of a perivalvular abscess. This case highlights risk factors for *C. striatum* infection and exemplifies the importance of recognizing this bacteria species as a possible pathogen causing complicated IE.

## Introduction

*Corynebacterium *species found in blood cultures may often be considered a contaminant due to its normal appearance on human skin and mucous membranes [1]. A retrospective population-based study assessed that of 335 episodes of *Corynebacterium *bacteremia, only 30 (8.8%) were a true infection [2]. There is a growing field of literature revealing the extent of *Corynebacterium *infection causing a variety of diseases including pulmonary and central nervous system infections [3]. This case demonstrates the pathogenicity and medical management of *C. striatum* endocarditis.

This article was previously presented as a poster at the SSM Health Saint Louis University Hospital Poster Palooza on June 18, 2024.

## Case presentation

A 58-year-old woman presented from a long-term acute care facility with two days of shortness of breath, abdominal distention, encephalopathy, and a fever of 103.0°F. The initial workup at the outside care facility revealed two positive blood cultures with *C. striatum*. The patient was empirically started on vancomycin, cefepime, and metronidazole after the development of septic shock and was intubated at the outside facility before transfer to our hospital. On admission, the patient was still febrile (100.2°F) and on a ventilator and pressor support. The initial physical exam of the patient was notable for open sores on her right foot and right leg with extensive cellulitis of her bilateral upper medial thighs with skin thickening and discoloration. The cardiac exam was unremarkable without murmurs.

The patient had a past medical history of heart failure with preserved ejection fraction, paroxysmal atrial fibrillation not on anticoagulation due to uterine bleeding, severe obesity, end-stage renal disease managed with hemodialysis through an internal jugular tunneled catheter, type 2 diabetes mellitus complicated by retinopathy and neuropathy, and infarct of right thalamus with residual left hemiparesis.

Initial differential diagnosis included bacteremia with septic shock with associated encephalopathy versus cardioembolic stroke from either bacterial endocarditis or atrial fibrillation. The source of infection may have originated from either cellulitis of the lower extremities, infection of chronic wounds on the lower extremities, or an infection of the internal jugular tunneled catheter placed prior to hospitalization.

**Figure 1:**
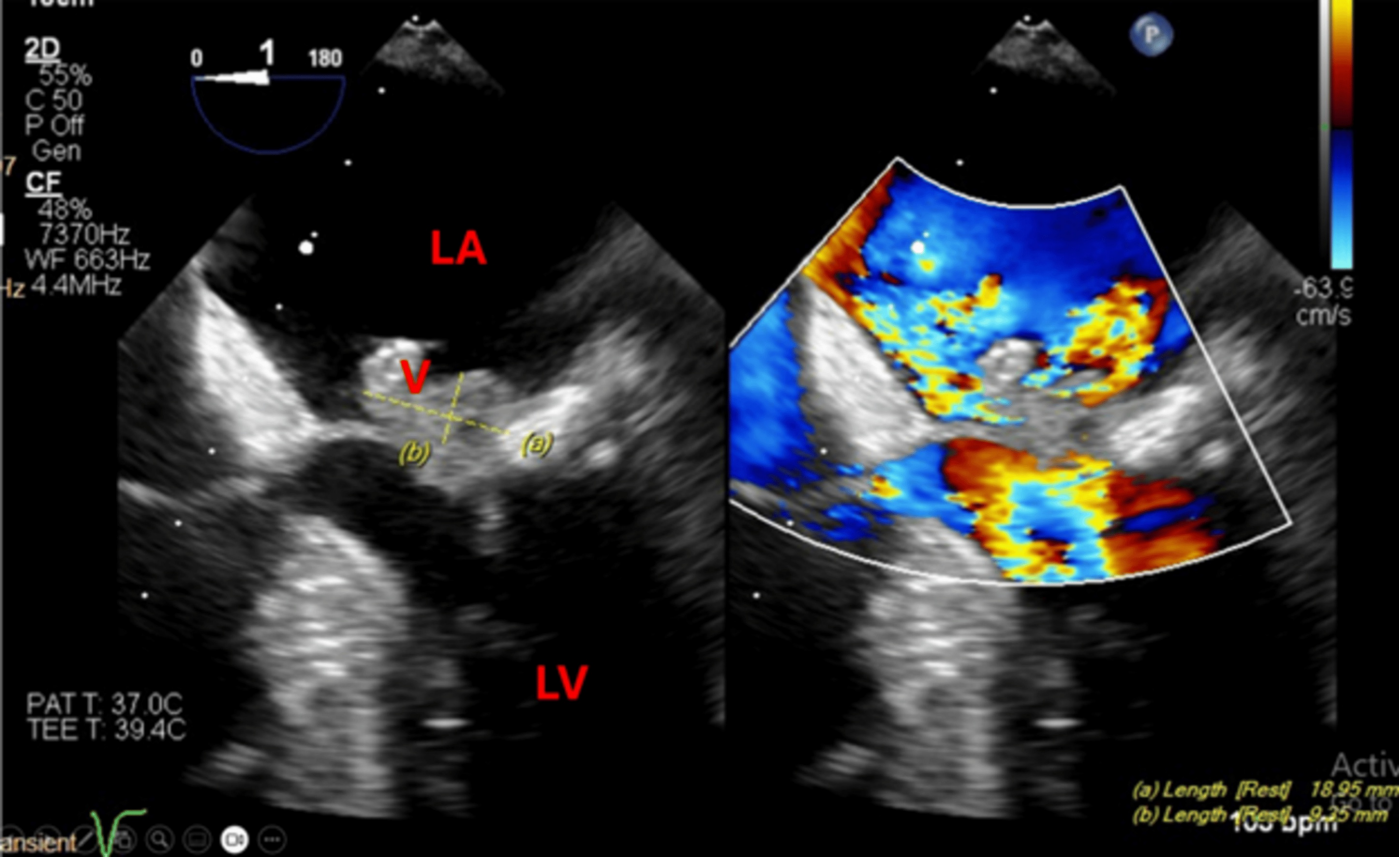
Two-chamber 2D mid-esophageal view (left) with color comparison (right) showing the vegetation on the mitral valve and multiple regurgitant jets. V: vegetation; LA: left atrium; LV: left ventricle

**Figure 2:**
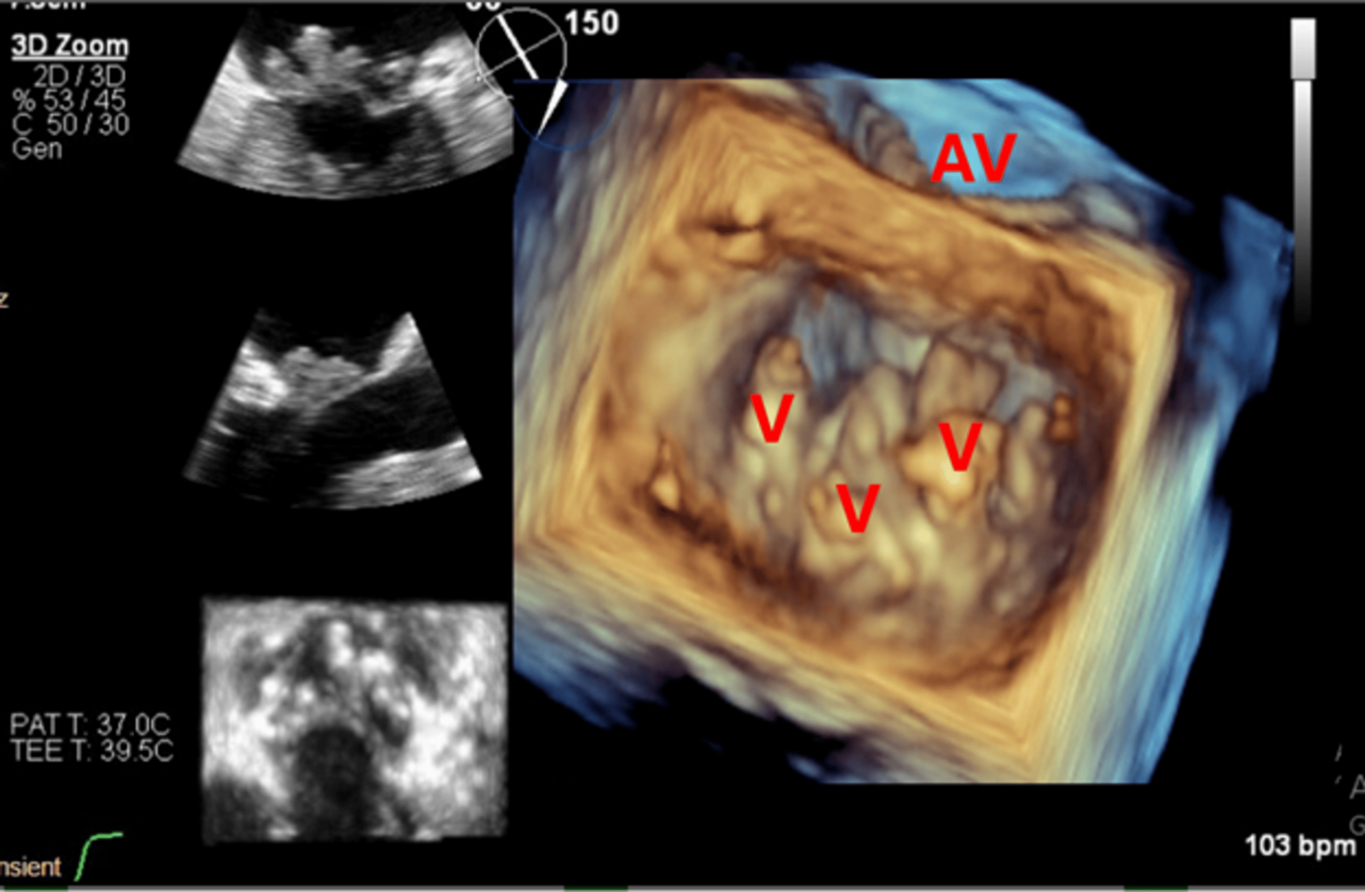
3D zoom of the mitral valve (surgeon’s view) showing the multilobed vegetation on the posterior leaflet. V: vegetation; AV: aortic valve

**Figure 3:**
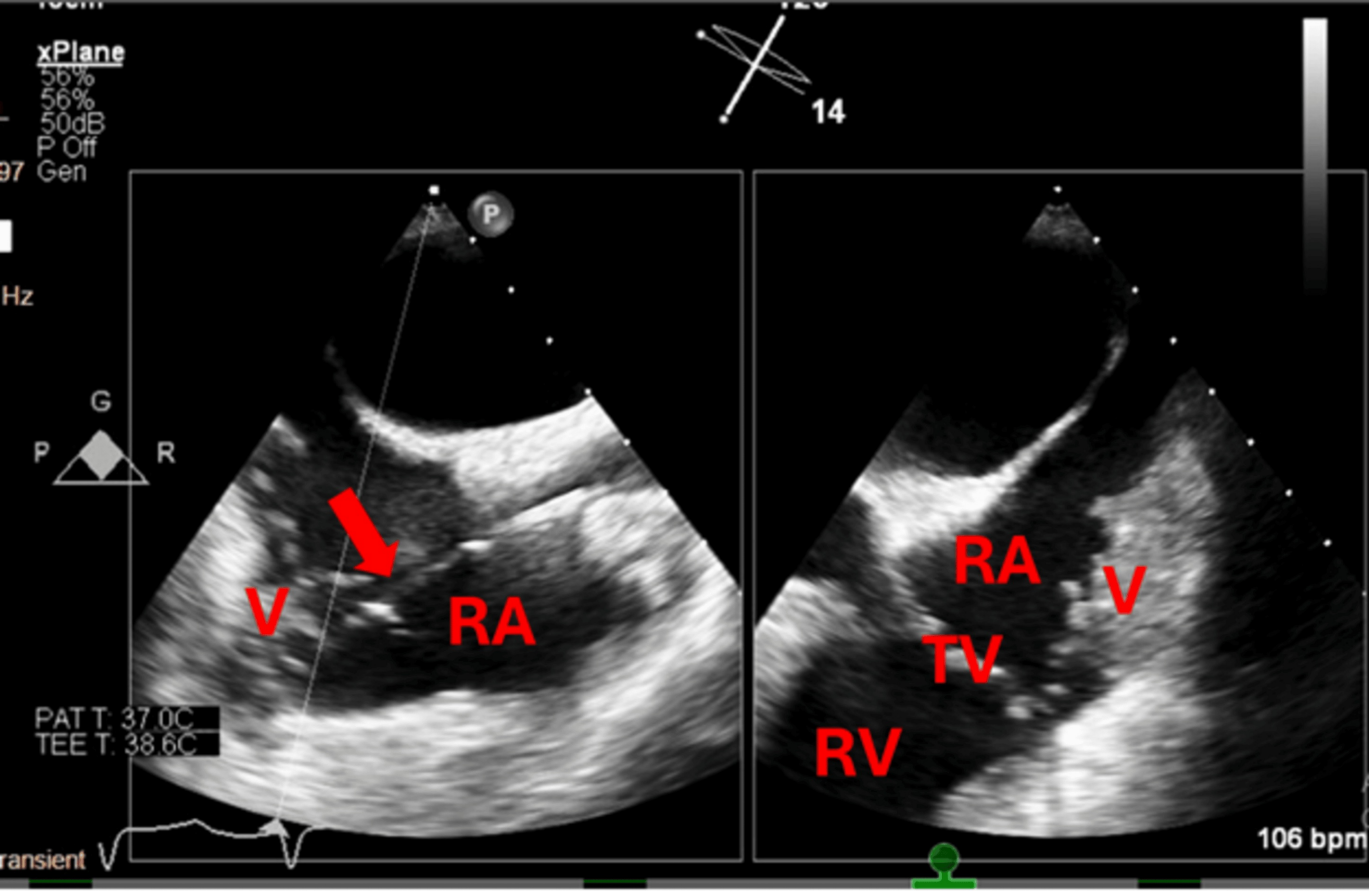
Bicaval view (left) with x-plane (right) showing the relationship of the catheter (red arrow) with the vegetation in the right atrium. V: vegetation; RA: right atrium; RV: right ventricle; TV: tricuspid valve

Initial laboratory investigations were positive for leukocytosis, anemia, elevated troponin-I, and elevated brain natriuretic peptide (Table 1). Repeat blood cultures completed 48 hours after initial cultures were negative. Initial transthoracic echocardiography (TTE) was poor quality but suggested mitral valve vegetation. Transesophageal echocardiography (TEE) performed on hospital day 3 identified severe mitral stenosis and regurgitation with a 2.2cm x 1.7cm partially mobile irregular mass present on the posterior leaflet of the mitral valve (Figures 1-2). Additionally, it identified a 2.2cm x 1.1cm right atrial mass on a peripherally inserted central catheter tip abutting the tricuspid valve concerning for a vegetation (Figure 3).

**Table 1:** Initial laboratory investigation

Laboratory Parameter	Result	Reference Range
White blood cell count (x10^9/L)	21.4	4.0-10.7
Hemoglobin (g/dL)	10.3	11.9-15.8
Brain natriuretic peptide (pg/mL)	1,492	<100
Troponin-I (ng/L)	858	<=14

Other imaging collected initially included computed tomography (CT) imaging of the abdomen, pelvis, and lower extremities which were notable for several splenic infarcts. CT head and CT angiography were performed which confirmed occlusion of the left posterior inferior cerebellar artery and acute infarct of the left cerebellum. Anticoagulation was held for concern of a hemorrhagic transformation of the patient’s stroke.

The patient was determined to not be a surgical candidate due to her high mortality risk from her extensive co-morbidities. Culture susceptibility results returned and showed susceptibility to vancomycin, linezolid, and gentamycin. Repeat blood cultures on hospital days 7 and 9 were positive for *Prevotella melaninogenica* and *Prevotella nanceienisis* respectively but with only one culture positive for each. The patient was restarted on metronidazole, and repeat blood cultures on hospital day 10 were negative. The final antibiotic regimen included vancomycin and metronidazole for a total of six weeks.

Repeat TTE on hospital day 30 was significant for the progression of mitral valve endocarditis with a larger posterior leaflet mobile vegetation on the mitral valve with a possible abscess in the posterior annulus with worsening mitral regurgitation and new aortic regurgitation.

The patient was discharged on hospital day 40 after completion of treatment of *Proteus mirabilis *pneumonia acquired during her hospitalization. She was discharged to a long-term acute care hospital for continued management of life-long antibiotics.

## Discussion

Non-diphtheroid *Corynebacterium *species are often disregarded as a contaminant in blood cultures but do uncommonly cause illness [4]. This case of *C. striatum *infective endocarditis (IE) demonstrates some principles documented in the literature but also highlights unique information about the pathogenicity of *C. striatum*, treatment therapies, and the importance of prompt initiation of therapy.

This immunocompetent patient was treated with vancomycin, for which *C. striatum* is reported to be universally susceptible, within 48 hours after the onset of symptoms [4]. Despite medical management, the patient continued to develop IE complications including perivalvular abscess and embolic strokes. These findings showing the severity of the disease are congruent with current literature with over 43% of patients with *Corynebacterium* IE ultimately succumbing to their illness. However, fewer patients (28%) needed a surgical intervention [5].

With this high chance of mortality and significant sequelae of infection, prudent management of positive *C. striatum *cultures is of paramount importance. Clinicians are faced with the challenge of separating true infection from contamination. There has been a report of delayed TEE imaging in a patient with true IE after multiple positive *Corynebacterium *cultures due to this uncertainty [6].

Assessing risk factors for *C. striatum* infection may help in diagnosis. Infection is often associated with invasive medical devices such as hemodialysis access, pacemaker leads, prosthetic valves, and other catheter tips [1,7]. *C. striatum *is known to form biofilms on a wide variety of medical device surfaces [8]. Studies suggest that 70% of *C. striatum* IE infections occur on prosthetic valves [9]. Other literature reviews show higher rates of prosthetic valve predominance with all reported cases occurring on prosthetic valves [2]. Another risk factor in this case is prolonged hospitalization, as described in the literature [8]. Over 50% of *C. striatum* infections occur as nosocomial infections [3].

## Conclusions

This case demonstrates an instance of IE likely caused by *C. striatum*. Higher suspicion of true infection may be called for in patients with invasive medical devices or when bacteremia is identified after prolonged hospitalization. This infection can be rapidly progressive and cause a significant disease burden. Timely identification of this historical contaminate as a true infection followed by treatment is essential for suppressing disease progression.
